# Cisplatin-Associated Ototoxicity: A Review for the Health Professional

**DOI:** 10.1155/2016/1809394

**Published:** 2016-12-27

**Authors:** Jessica Paken, Cyril D. Govender, Mershen Pillay, Vikash Sewram

**Affiliations:** ^1^Discipline of Audiology, School of Health Sciences, University of KwaZulu-Natal, Private Bag X54001, Durban 4000, South Africa; ^2^African Cancer Institute, Faculty of Medicine and Health Sciences, Stellenbosch University, P.O. Box 241, Cape Town 8000, South Africa; ^3^Division of Community Health, Faculty of Medicine and Health Sciences, Stellenbosch University, P.O. Box 241, Cape Town 8000, South Africa

## Abstract

Cisplatin is an effective drug used in the treatment of many cancers, yet its ototoxic potential places cancer patients, exposed to this drug, at risk of hearing loss, thus negatively impacting further on a patient's quality of life. It is paramount for health care practitioners managing such patients to be aware of cisplatin's ototoxic properties and the clinical signs to identify patients at risk of developing hearing loss. English peer-reviewed articles from January 1975 to July 2015 were assessed from PubMed, Science Direct, and Ebscohost. Seventy-nine articles and two books were identified for this review, using MeSH terms and keywords such as “ototoxicity”, “cisplatin”, “hearing loss”, and “ototoxicity monitoring”. This review provides an up-to-date overview of cisplatin-associated ototoxicity, namely, its clinical features, incidence rates, and molecular and cellular mechanisms and risk factors, to health care practitioners managing the patient with cancer, and highlights the need for a team-based approach to complement an audiological monitoring programme to mitigate any further loss in the quality of life of affected patients, as there is currently no otoprotective agent recommended routinely for the prevention of cisplatin-associated ototoxicity. It also sets the platform for effective dialogue towards policy formulation and strengthening of health systems in developing countries.

## 1. Introduction

Cancer places a huge burden on society and has been identified as the leading cause of death in both more and less economically developed countries [1]. Projections based on the GLOBOCAN 2012 estimates predict a substantive increase to 19.3 million new cancer cases per year by 2025, due to growth and ageing of the global population. South Africa, like other developing countries, is also experiencing an increase in the overall burden of disease attributable to cancer, with the number of new cancer cases predicted to increase by 46% by 2030 [2]. This is likely to result in an increase in the use of cancer chemotherapy agents, which assist in preventing the proliferation, invasion, and metastases of the cancer cells [3].

The basis for chemotherapy is anticancer drugs containing platinum, that is, cisplatin (cis-diamminedichloroplatinum II) and carboplatin (cis-diammine 1,1-cyclobutane dicarboxylatoplatinum II) [4]. Other chemotherapy drugs include nitrogen mustard, amino-nicotinamide, dichloromethotrexate, bleomycin, and 5-fluorouracil [5, 6]. The first of these drugs, that is, cisplatin, consists of a divalent Pt (II) central atom and four ligands of cis-positioned pairs of chlorine atoms or amine groups [3].

Since its discovery in the 1970s [7], cisplatin continues to be hailed as one of the most potent cancer chemotherapeutics in children and adults, as it is unique and unmatched in its effectiveness against many cancers [4], namely, osteogenic sarcoma, medulloblastoma, testicular, cervical, and ovarian cancers [8]. Similarly, its toxicity profile is expansive, involving the gastrointestinal, hematologic, renal, and auditory systems [8]. While the use of saline hydration and mannitol diuresis may prevent nephrotoxicity, neurotoxicity is still not curable or preventable [9].

Ototoxicity refers to the hearing disorder that results from the temporary or permanent inner ear dysfunction after treatment with an ototoxic drug [10]. Other drug classes known to have ototoxic properties include aminoglycosides, loop diuretics, quinine, nonsteroidal anti-inflammatory drugs [11], and antiretroviral therapy (ART) [12]. This is of concern in South Africa, as it is estimated that 12.2% of the population (6.4 million persons) were HIV positive in 2012, which is 1.2 million more people living with HIV than in 2008 (10.6%, or 5.2 million) [13]. Resultantly, ART exposure had almost doubled from 16.6% in 2008 to 31.2% in 2012 [13]. Not only will many infected people be at risk for ototoxicity due to ARTs, but a large number will also be susceptible to HIV-related cancers, such as Kaposi's sarcoma, Non-Hodgkin's lymphoma, and cervical cancer, as well as infectious diseases such as tuberculosis, conditions that often require pharmacological therapy with the adverse side effect of ototoxicity. It is possible that their treatments could consist of simultaneous use of more than one ototoxic drug, increasing the likelihood of ototoxicity. All health care professionals managing patients with cancer should therefore be knowledgeable about the ototoxic properties of cisplatin.

However, Malhotra [7] indicated that most oncologists in India do not make referrals for audiological evaluations of patients receiving cisplatin, while a study in South Africa revealed that the effects of ototoxicity, the role of audiologists, and need for their expertise were not fully realized by the oncologists sampled [14]. This is further supported by evidence from the South African study of Khoza-Shangase and Jina [15] which indicated that most general practitioners sampled also do not appear to carry out ototoxicity monitoring strategies, despite being aware of their own role within an ototoxicity monitoring programme. This review therefore aims to serve as resource for health professionals to enhance their understanding of ototoxicity as well as their roles within an ototoxicity monitoring programme by providing an overview and description of this condition in patients diagnosed with cancer and receiving cisplatin chemotherapy.

## 2. Method

The review identified peer-reviewed articles available from January 1975 to July 2015 on the topic of cisplatin-associated ototoxicity and ototoxicity monitoring and included English articles only. The same researcher conducted the literature search and reviewed the abstracts and articles for inclusion in the study. Studies were identified using keyword and MeSH term searches of electronic databases depicted in Table 1. A manual search of relevant authors and journals was also completed. The references cited by each publication, review, or book chapter were reviewed in order to locate additional potential publications.

In order to be selected, the article had to present data on either cisplatin ototoxicity and/or ototoxicity monitoring in human participants, and no research designs were excluded. Running these searches yielded a total of 2106 records, of which 1581 were excluded based on the title and/or abstract as well as duplication. Eighty-five relevant articles, comprising six national and 79 international articles, were selected. Information was also obtained from four internationally published books. A perusal of narrative reviews of other auditory pathologies was conducted in an attempt to determine areas of significance for an overview of cisplatin ototoxicity. This resulted in the following eight areas being included: the mechanisms of cisplatin ototoxicity, clinical presentation, risk factors, incidence rates in adults and children, the effect on quality of life, ototoxicity monitoring, otoprotective strategies, and management of an ototoxic hearing loss.

### 2.1. The Mechanisms of Cisplatin Ototoxicity

**Figure 1:**
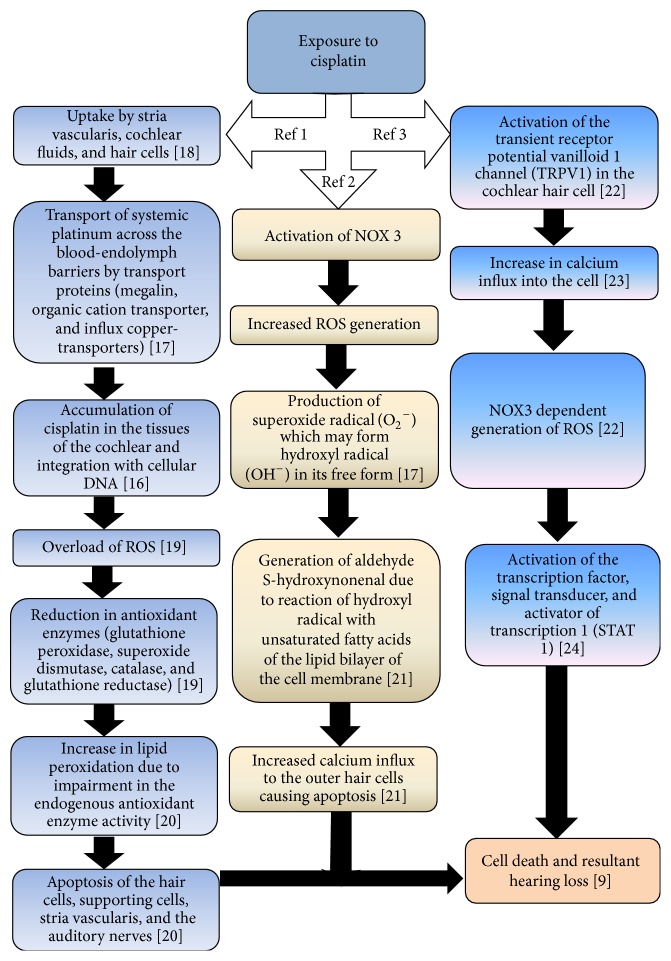
Mechanisms of cisplatin ototoxicity REF 1 [16–20], REF 2 [17, 21], and REF 3 [22–24].

Cisplatin ototoxicity is produced by several distinct mechanisms [16] as depicted in Figure 1. One such mechanism, the antioxidant model, involves the formation of reactive oxygen species (ROS) within the cochlea and consequent reduction in antioxidant enzymes following exposure to cisplatin chemotherapy [16–20]. Another mechanism of cisplatin ototoxicity involves the significant contribution of nicotinamide adenine dinucleotide phosphate oxidase 3 isoform (NOX3) to the generation of reactive oxygen species within the cochlea, when activated by cisplatin [17, 21], while a third mechanism relates to the activation of transient receptor potential vanilloid 1 channel (TRPV1) [22–24].

The molecular mechanisms of cisplatin ototoxicity therefore include the following:

- “Creation of reactive oxygen species,
- Depletion of antioxidant glutathione and its regenerating enzymes,
- Increased rate of lipid peroxidation,
- Oxidative modifications of proteins,
- Nucleic acids damage by caspase system activation and
- S-Nitrosylation of cochlear proteins” [25].

With the cellular mechanisms of cisplatin-associated ototoxicity including damage to the outer hair cells, supporting cells, marginal cells of the stria vascularis, spiral ligament, and the spiral ganglion cells [25], it is evident that the structures of the inner ear are most susceptible to damage by cisplatin chemotherapy, with apoptotic degeneration of the hair cell in the organ of Corti being most prominent [26]. The outer hair cells in the basal turn of the cochlea are most affected [27, 28]. This leads to an initial elevation of high frequency audiometric thresholds, followed by a progressive loss into the lower frequencies with continued therapy [27, 28]. Knowledge of the different mechanisms of cisplatin ototoxicity is important for health care professionals as it will create an awareness of its complexity and the resulting clinical presentation.

### 2.2. Clinical Presentation and Risk Factors

Cisplatin-associated ototoxicity usually manifests as irreversible, progressive [8], bilateral, high frequency sensorineural hearing loss [29] with tinnitus [30]. The latter may occur with or without a hearing loss [29] and may be permanent or transient, sometimes disappearing a few hours after treatment [31] or alternatively persisting a week after treatment [32]. While most of the hearing loss is permanent, there is sometimes sporadic and partial recovery [31]. In addition, rare cases of unilateral hearing loss have been reported, which are usually explained by tumour location and surgical or therapeutic intervention on the affected side [33]. Moreover, the hearing loss is not always symmetrical [33, 34], with Jenkins et al. [34] finding that 75% of women on cisplatin chemotherapy displayed an asymmetry of hearing thresholds of at least 10 dB between ears posttreatment. Schmidt et al. [33], in their investigation of 55 children on cisplatin chemotherapy, found that the high frequency hearing thresholds were slightly elevated in the left ear and that males had a greater degree of hearing loss than the females.

The degree of hearing loss is often variable and is related to the dose; that is, the higher the cumulative dose, the greater the ototoxic effect [35, 36]. The duration, number of cycles administered [37], and method of administration [38] also influence cisplatin-associated ototoxicity. Additional factors that may increase ototoxicity include exposure to concomitant noise [39], chemicals, and other ototoxic medications [35]. Furthermore, evidence also shows that melanin content is related to an increased risk of cisplatin-associated ototoxicity [40]. Individuals with dark eyes and therefore a higher melanin content in the cochlear are at greater risk of ototoxic damage, as the melanin causes retention of the platinum within the cochlear and subsequently increases the risk of damage [41, 42]. Individuals presenting with renal insufficiency, that is, high levels of serum creatinine, are at a greater risk for cisplatin-associated ototoxicity [35]. Genetic risk factors, such as megalin and glutathione S-transferases gene polymorphism, have also been reported to influence cisplatin ototoxicity [43], as do physiological factors such as age, with younger children [44] and older adults (older than 46 years) [45] presenting with a greater severity of hearing damage. Preexposure hearing ability may also impact on incidence rates [35, 46]. Awareness of these risk factors may assist health care professionals with informational counselling of the patient receiving cisplatin chemotherapy.

### 2.3. Cisplatin-Associated Hearing Loss in Adults and Children

The incidence of cisplatin ototoxicity is variable in adults (Table 2) and children (Table 3). The variations may be due to a number of factors, such as differences in the dose, both within a cycle and the total amount administered over multiple cycles, time interval between courses, method of administration, and treatment duration, as well as differences in patient population. Further exploration in this regard is therefore necessary.

### 2.4. Quality of Life

Ototoxicity poses a major problem to the cancer patient, as the quality of life after receiving cisplatin chemotherapy may be negatively affected due to hearing loss, resulting in social, emotional, and vocational difficulties, as effective communication is often hindered. Tasks that normal hearing persons take for granted may become challenging and frustrating [58]. In addition, an individual's safety may be compromised due to the hearing loss, as appropriate response to alarms and warning signals may be delayed. Furthermore, a hearing loss may also result in psychosocial and physical health problems, as well as depression and social isolation [59]. Hence, hearing loss, often referred to as the “invisible condition,” has serious visible ramifications on the quality of life of a hearing impaired individual [58]. This is particularly relevant if the individual has already experienced the hearing world, as the hearing function is never restored to normal, even though patients may benefit from the use of assistive listening devices, such as hearing aids and cochlear implants [10].

The impact of an ototoxic hearing loss may be more profound for infants and young children who are at a critical stage of their speech and language development [60]. Furthermore, the high frequency nature of an ototoxic hearing loss may result in speech recognition and comprehension being compromised [61], resulting in possible neurocognitive and psychosocial delays [62]. There is also an elevated risk for academic learning problems and psychosocial difficulties in school-aged children and adolescents [63]. Literature indicated that childhood survivors of neuroblastoma had twice the rate of difficulties, as indicated by parent reports, with reading and math skills, and/or attention and a higher risk of a general learning disability than those without a hearing loss. There was also poorer self-reported quality of life scores in these children with regard to school functioning [63]. Hence, cisplatin-associated ototoxicity further complicates the morbidity of cancer patients [8], as it would also isolate them from family members and significant others at a time when they require the greatest support.

### 2.5. Ototoxicity Monitoring

Advancements in medical knowledge and technology, such as screening and early detection of several cancers, have resulted in notable improvements in relative five-year survival rates for cancer [64, 65]. Therefore, improving the quality of life after cisplatin-based chemotherapy becomes increasingly important, and resulting comorbidities such as ototoxicity can be managed appropriately and immediately [14] if adequate monitoring is in place.

The nature of ototoxicity is such that it often goes undetected until speech intelligibility is affected [66] and is usually detected when a communication problem becomes evident [67]. Communication problems, such as constantly asking for repetition or not responding when spoken to, signify that the hearing loss has progressed to the frequencies important for understanding speech [67]. In this case, an audiological monitoring programme can avert, to a large extent, the reduced quality of life as a result of hearing loss, as patients on cisplatin chemotherapy can be identified early, counselled, monitored, and managed appropriately through interventions in a logical, systematic, and coherent manner.

Audiological monitoring should aim to identify the hearing loss early and reduce its impact on the individual's life by means of proper medical and hearing intervention [68]. Prospective audiological evaluations remain the only reliable method for detecting ototoxicity before it becomes symptomatic [69]. An ototoxicity monitoring programme should involve a health care team comprising of an oncology nurse, oncologists, audiologist, and pharmacist to ensure effective sustainability of such a programme, if implemented, with the patient being the central focus. The audiologist is involved in identifying an ototoxic hearing loss, informing the oncologist of such a development, counselling the patient and their family, and prescribing amplification devices, such as hearing aids and cochlear implants [70]. Early identification of an ototoxic hearing loss provides oncologists with an opportunity to adjust the chemotherapy regimen in order to reduce or prevent further deterioration of hearing [70]. The oncologist and nurses should also counsel patients on the side effects of cisplatin, including ototoxicity, in an attempt to prepare them for treatment outcomes and help them set realistic expectations [71]. Pharmacists who have access to a patient's medication list may also alert the oncologists and audiologists to those who are on other ototoxic medication and therefore at a greater risk for cisplatin-induced ototoxicity. Effective management of such patients using evidence-based practices may improve management of those with cancer [72], ensuring that they and their families are counselled and appropriate interventions are timeously implemented. The principles of early identification and early intervention are a part of ototoxicity monitoring, and the audiologist can manage such a programme [56].

**Figure 2:**
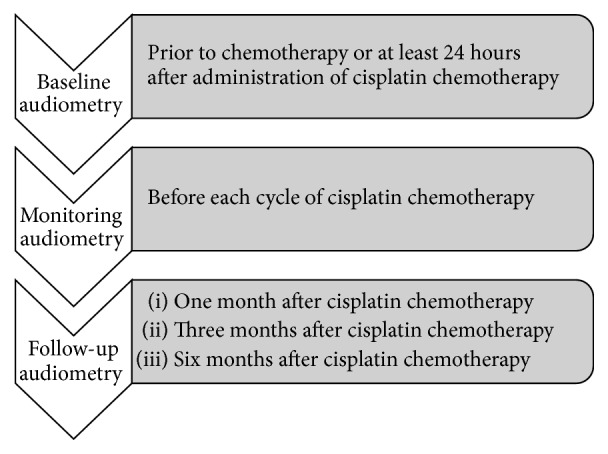
Timelines for audiological assessments [69].

In countries without ototoxicity management guidelines, the “Guidelines for the Audiological Management of Individuals receiving Cochleotoxic Drug Therapy” developed by the American Association of Speech-Language-Hearing Association [69] may, consequently, guide the audiologist in the implementation of an ototoxicity monitoring programme within a local, regional, or national setting. For widespread acceptance and use, ototoxicity monitoring programmes need to incorporate efficient and cost-effective ototoxicity identification techniques [67], while considering the health care system and demographics of the patient population being managed. For any population receiving ototoxic medication, the following should be considered: “(1) the patient's level of alertness or ability to respond reliably; (2) the most appropriate times during the treatment protocol for test administration, and; (3) the test should comprise the baseline, monitoring and post-treatment evaluations” [73]. Appropriate time intervals for audiological assessments may differ depending on the type of cancer as well as the frequency and dose of cisplatin (Figure 2) [69].

The audiological assessments should incorporate a detailed case history, otoscopic examination, immittance audiometry, speech audiometry, DPOAEs, and conventional and extended high frequency audiometry (i.e., up to 20 000 Hz) (HFA) [69, 73]. These procedures are all conducted for the baseline assessment and the six-month follow-up evaluation [69, 73]. While auditory brainstem response may be used, it is not considered a standard procedure for monitoring ototoxicity [73].

Monitoring audiological evaluations during treatment and the one- and three-month follow-up evaluations include case interview, otoscopy, and immittance audiometry as well as air conduction pure tone and objective testing [73]. However, full-frequency threshold testing is impractical for many patients on cisplatin chemotherapy, as these individuals are often extremely ill and easily fatigued. The use of abbreviated threshold monitoring procedures that are clinically practical for these patients is therefore recommended. One such method involves the use of the sensitive range for ototoxicity (SRO). This is “the highest frequency with a threshold at or below 100 dB SPL followed by the next six lower adjacent frequencies in 1/6-octave steps or the one octave range near the highest audible frequency” [73]. SRO is usually determined during the baseline evaluation and is dependent on each patient's hearing threshold configuration. During monitoring evaluations, air conduction thresholds should be determined within the patient's defined SRO. However, full-frequency testing should be conducted within the same session if an ASHA significant hearing change is noted within the SRO [69].

If a patient on cisplatin chemotherapy is still responsive and alert, the protocol presented above would be suitable. However, a patient who has limited responsiveness may be required to undergo the same audiological evaluations, except speech audiometry. Patients who are responsive as well as those who have limited responses can undergo both behavioural and objective testing. However, those patients who are too ill or too young to respond should undergo only objective testing, such as otoscopy, tympanometry, acoustic reflexes, and DPOAEs or ABRs [73].

While pure tone audiometry in the conventional frequency range is suitable for evaluating hearing in the range responsible for speech understanding, as well as for differential diagnosis, it is less sensitive to detecting early ototoxic change [11, 70]. The two tests identified as being the most important for the early detection of cisplatin ototoxicity are HFAs and OAEs, each also having limitations (see Table 4) [70]. Therefore, using each test in isolation may not be as effective as utilizing a test battery approach, as it increases the chances of obtaining reliable audiologic monitoring data over time. In addition, these two tests could be used to complement one another in every cycle of chemotherapy to ensure the earliest detection of ototoxicity [74].

The ototoxicity monitoring protocol proposed by ASHA [69] represents an aggressive and ideal approach for monitoring ototoxicity and is dependent on a country's infrastructure and resource constraints. The ASHA [69] guidelines may therefore not be generalized to a country without considering the contextual factors that may influence its applicability to that country. However, it does provide guidance towards creating a roadmap that countries, such as South Africa, may aspire towards in implementing an ototoxicity monitoring programme. Similar to India [79], no programmes have been formally implemented to identify and monitor ototoxicity in patients on cancer chemotherapy in South Africa. As a result, there is no contextually relevant research to steer the implementation of an accountable and effective ototoxicity monitoring program in the country. This is probably one of the main reasons for ototoxicity monitoring programmes not being commonplace in local hospitals and clinics. In addition, the health of South Africans is characterized by a quadruple burden of disease, encompassing the occurrence of infectious diseases, the rise of noncommunicable diseases, and perinatal and maternal disorders, as well as injuries and violence [80], which may result in cancer receiving low priority for health care services. However, the creation of an audiological monitoring programme allows for better control of cancer related comorbidities.

### 2.6. Otoprotective Strategies

Over the years, a number of studies have investigated the use of otoprotectants with cisplatin, their purpose being to protect the inner ear from any injury while not interfering with the antitumor effects of cisplatin [61]. Otoprotective strategies include reducing the formation of free radicals by maintaining glutathione levels and antioxidant activity [27]. Three mechanisms may provide protection against cisplatin, these being endogenous molecules, exogenous agents, or a combination of exogenous agents that trigger endogenous protective mechanisms. However, endogenous agents are not effective against cisplatin when the dose exceeds a certain threshold [17, 81].

Nearly all of the otoprotective agents are sulfur- or sulfhydryl-containing compounds (thio compounds), known as antioxidants, and potent heavy metal chelators [82]. The numerous otoprotective agents utilized in clinical and animal studies include Amifostine, d-or l-Methionine, methylthiobenzoic acid, lipoic acid, tiopronin, glutathione ester, sodium thiosulfate [83], Melatonin [84], Vitamin E [85], N-acetylcysteine [86], Dexamethasone [87], and Resveratrol [88]. However, none of these agents have been found to be unequivocally beneficial in preventing cisplatin ototoxicity and no agent is currently recommended for routine use [7]. Further research is therefore needed to find new methods and optimize old ones to prevent and/or treat hearing loss during cisplatin therapy. In addition, intratympanic administration of medication together with gene therapy needs to be further explored [25]. Intratympanic administration involves the diffusion of the otoprotective agent across the round window into the inner ear, where its therapeutic effect is exerted. An advantage of this method of administration is that there are higher concentrations of the otoprotective agent in the inner ear, this being in comparison to the use of oral or parenteral administration, without potentially reducing the efficacy of the cisplatin treatment [89, 90]. The disadvantage of this procedure, however, is that each ear would have to be treated with a moderately invasive procedure [91]. Alternatively, gene therapy may prove to be beneficial in protecting an individual against cisplatin-induced hearing loss as several genes, namely, megalin, glutathione-S-transferases, Thiopurine* S*-methyltransferase, and catechol-*O*-methyl transferase, may be responsible for susceptibility to hearing loss [9].

### 2.7. Management of an Ototoxic Hearing Loss

If a cisplatin-associated hearing loss results in communication difficulties, it is the audiologist's ethical responsibility to begin or recommend aural rehabilitation [69]. However, this intervention should not only occur once hearing loss has been detected but before the patient begins the cisplatin chemotherapy. Aural rehabilitation techniques such as speech reading and counselling on compensatory communication strategies should be conducted. The counselling should include spouses and significant other, as hearing loss may not only impact the person with cancer but also frequent communication partners [92]. Patients with sensorineural hearing loss due to the use of cisplatin may also benefit from the use of assistive listening devices such as hearing aids or cochlear implants [10]. Children with ototoxic hearing loss may also require the use of personal frequency modulated systems in the classroom.

Furthermore, with the recent developments in hearing aid technology, a patient with an ototoxic hearing loss is more likely to receive the desired amplification benefit. These developments in technology include

- “Extended bandwidth” hearing aids. These hearing aids are able to amplify sounds at and above 8000 Hz. However, there is limited data indicating significant improvements in speech recognition with the use of this technology [93].
- Hearing aids with frequency lowering technology achieved by linear frequency transposition, nonlinear frequency compression, or spectral envelope warping. Frequency lowering is used to overcome the limits of either the bandwidth of the device or the functional bandwidth of the ear, by lowering high frequency energy to a region that is more likely to provide and/or benefit from audible sound [94]. While there are no published studies suggesting one approach to be superior to another, frequency lowering technology has been found to improve audibility and speech understanding of high frequency sounds [93]. Commercially available types of frequency lowering signal processing include frequency transposition (Widex), nonlinear frequency compression (Phonak), and frequency translation (Starkey). These processors are commercially labelled as Audibility Extender, SoundRecover, and Spectral IQ, respectively [94].

## 3. Conclusion

This review has highlighted that cisplatin ototoxicity is a frequent adverse event of cisplatin chemotherapy that may negatively affect the quality of life of patients with cancer. The different molecular and cellular mechanisms involved in cisplatin-associated ototoxicity highlight the complexity of this condition and the consequent difficulty in identifying an effective otoprotective agent. The varying incidence rates reported in both adults and paediatrics may be due to the different audiological tests employed in the monitoring of the cancer patient's hearing status and therefore highlight the importance of the use of extended high frequency audiometry and DPOAEs in ototoxicity monitoring. An audiological monitoring programme comprising a team of health care professionals, knowledgeable about cisplatin ototoxicity, may therefore serve to improve evidence-based service delivery to these patients.

## Figures and Tables

**Table 1:** Search and MeSH terms used in the literature search.

Electronic database	Search term	MeSH term
PubMed (Medline)	Ototoxicity [All Fields] AND monitoring [All Fields]	((“cisplatin” [MeSH Terms] OR “cisplatin” [All Fields]) AND ototoxicity [All Fields]) OR ((“cisplatin” [MeSH Terms] OR “cisplatin” [All Fields]) AND (“hearing loss” [MeSH Terms] OR (“hearing” [All Fields] AND “loss” [All Fields]) OR “hearing loss” [All Fields]))

Science Direct		“cisplatin ototoxicity” or “cisplatin hearing loss” “ototoxicity monitoring”

Ebscohost	Cisplatin ototoxicity or cisplatin hearing loss Ototoxicity monitoring	

**Table 2:** Studies reflecting cisplatin-associated hearing loss in adults.

Study	Country	Type of study	Audiological tests conducted	Patient population	Number of patients who developed ototoxicity
Malgonde et al. [47]	India	Prospective	Pure tone audiometry (frequencies not specified) and short increment sensitivity index test	34 patients with head and neck cancers receiving cisplatin containing chemotherapy and concomitant radiation therapy	34 (100%)

Whitehorn et al. [48]	South Africa	Retrospective cross-sectional	Air (0.25–8 kHz) and bone conduction pure tone audiometry	107 patients receiving cisplatin containing chemotherapy, irrespective of the type of the cancer	59 (55.1%)

Nitz et al. [49]	Germany	Prospective longitudinal trinational population-based	Air (0.125–8 kHz) and bone conduction pure tone audiometry	1 patient with soft-tissue sarcoma and 16 with osteosarcoma, receiving cisplatin and/or carboplatin containing chemotherapy	6 (35.3%)

Arora et al. [8]	India	Prospective, randomized, observational	Pure tone air (0.25–16 kHz) and bone conduction audiometry Results are reflective of frequencies 4 to 16 kHz.	57 patients receiving cisplatin containing chemotherapy:	—
				10 patients (low dose group, carcinoma of the larynx)	6 (60%)
				35 patients (middle dose group, head and neck cancers, carcinoma of the cervix)	35 (100%)
				12 patients (high dose group, carcinoma of the lung and carcinoma of the testis)	12 (100%)

Dell'Aringa et al. [50]	Brazil	Case series	Tympanometry, acoustic reflex threshold testing, distortion product otoacoustic emissions (DPOAEs), air (0.25–8 kHz) and bone conduction pure tone audiometry, speech audiometry	17 patients with extracranial head and neck cancers receiving cisplatin containing chemotherapy and concomitant radiation therapy	12 (70.5%), left ears; 11 (64.7%), right ears

Schultz et al. [51]	Brazil	Prospective	Full audiometric evaluations, with only air (0.25–8 kHz) and bone conduction pure tone audiometry thresholds computed	31 patients receiving cisplatin containing chemotherapy, irrespective of the type of cancer	12 (38%), NCI criteria; 19 (65%), Brock et al.'s criteria; 17 (54%), ASHA criteria; 9 (29%), David and Silverman's criteria

Zuur et al. [52]	The Netherlands	Prospective	Air (0.125–16 kHz) and bone conduction pure tone audiometry	60 patients with locally advanced head and neck cancer, receiving cisplatin containing chemotherapy and concomitant radiation therapy	19 (31%), up to 8 kHz; 28 (47%), up to 16 kHz

Dutta et al. [36]	India	Prospective	Pure tone audiometry (frequencies not specified)	60 patients receiving cisplatin containing chemotherapy, type of cancer not indicated	9 (15%)
				51, low dose group	6 (12%)
				9, high dose group	3 (33%)

Strumberg et al. [53]	Germany	Retrospective	Pure tone air (0.125–12 kHz) and bone conduction audiometry, transient evoked otoacoustic emissions test (TEOAE)	32 patients with testicular cancer receiving cisplatin containing chemotherapy	21 (70%)

Nagy et al. [54]	USA	Retrospective	Tympanometry, air (0.25–8 KHz) conduction pure tone audiometry	53 patients with oesophageal, lung, or head and neck cancer receiving cisplatin containing chemotherapy and concomitant radiation therapy (only for head and neck cancer)	19 (36%)

Bokemeyer et al. [35]	Germany	Retrospective	Pure tone air (0.5–8 kHz) and bone audiometry	86 patients with testicular cancer receiving cisplatin containing chemotherapy	57 (66%)

Waters et al. [32]	Canada	Retrospective	Pure tone air (0.25–8 kHz) and bone conduction audiometry, immittance audiometry, and speech audiometry	60 patients with advanced ovarian carcinomas receiving cisplatin containing chemotherapy	
				39, low dose, short treatment (25 from LDE group and 14 new cases after treatment modification)	6 (15%)
				8, low dose, blocks	0 (0%)
				25, low dose, extended treatment	9 (36%)
				13, high dose, short treatment	12 (92%)

**Table 3:** Studies reflecting cisplatin-associated hearing loss in children.

Study	Country	Type of study	Audiological tests conducted	Patient population	Number of patients who developed ototoxicity
Nitz et al. [49]	Germany	Prospective longitudinal trinational population-based	Air (0.125–8 kHz) conduction pure tone audiometry	93 patients with osteosarcoma and 19 with soft-tissue sarcoma receiving cisplatin and/or carboplatin containing chemotherapy	55 (49.1%)

Knight et al. [55]	USA	Prospective	Otoscopy, tympanometry, pure tone audiometry (0.5–8 kHz), DPOAEs, and ABR	32 children with different types of cancers treated with cisplatin and/or carboplatin containing chemotherapy	20 (62.5%)
			Otoscopy, tympanometry, extended pure tone audiometry (0.5–16 kHz), and DPOAEs	17 children with different types of cancers treated with cisplatin and/or carboplatin containing chemotherapy	16 (94.1%)

Coradini et al. [44]	Brazil	Retrospective	Tympanometry, pure tone audiometry (0.25–8 kHz), TEAOEs, and DPOAEs	23 children with malignant hepatic tumour, osteosarcoma, and germ cell tumours receiving cisplatin containing chemotherapy	12 (52%), pure tone; 5 (22%), TEOAEs; 16 (71%), DPOAEs

Bertolini et al. [56]	France	Prospective	Otoscopy, immittance audiometry, speech audiometry, play audiometry or free-field audiometry, conventional pure tone audiometry (frequencies not specified), or ABR (depending on the age of the participant)	102 children with either neuroblastoma, hepatoblastoma, germ cell tumour, or osteosarcoma	—
				96 received cisplatin and/or carboplatin containing chemotherapy	39 (41%)
				52 received cisplatin only	19 (37%)

Stavroulaki et al. [57]	Greece	Prospective	Otoscopy, immittance audiometry, pure tone audiometry (0.25–8 kHz), TEOAEs, and DPOAEs	12 children with either neuroblastoma, osteosarcoma, medulloblastoma, rhabdomyosarcoma, or primitive neuroectodermal tumour receiving cisplatin containing chemotherapy	6 (50%)

**Table 4:** Clinical significance and limitations of HFA and OAEs.

HFA (>8kHz)	OAEs
*Clinical significance for ototoxicity*	
(i) HFA is considered to be the most sensitive test to identify ototoxic hearing loss [8, 55, 75].	(i) OAES is considered a noninvasive objective measure of cochlear outer hair cell function [76].
(ii) HFA is not as affected by middle ear pathologies as OAEs [11].	(ii) DPOAEs can be regarded as a more sensitive measure for the early detection of hearing loss than conventional pure tone audiometry [44].
(iii) The criteria of change for ototoxicity is established [11].	(iii) OAEs is time efficient [11].
	(iv) DPOAEs provide frequency specific information [67].

*Limitations*	
(i) HFA is not standardised [11].	(i) OAEs are significantly affected by middle ear pathology [37].
(ii) HFA is not commonly used, due to the need for additional equipment such as circum-aural headphones [77].	(ii) There is no universal value for the criteria of change indicating ototoxicity [76].
(iii) HFA may not always be applicable, as patients with hearing loss in the conventional frequency range may not have measurable hearing in the extended high frequency range [78].	(iii) OAEs are absent in patients with moderate degrees of hearing loss [67].
(iv) Test efficiency may be affected due to HFA being time consuming [70].	(iv) OAEs have a limited frequency range (generally up to 8000 Hz) [67].
